# Herbert William Allingham

**Published:** 1905-01

**Authors:** 


Herbert William Allingham, F.R.C.S., senior assistant surgeon of Saint George's Hospital, London, died at Marseilles, November 4, 1904, aged 42 years. Mr. Allingham was surgeon to His Majesty's household, surgeon in ordinary to His Royal Highness, the Prince of Wales, honorary surgeon to King Edward VII. Hospital for Officers, and to the Osborne Home for Officers. He also held several other hospital appointments; and, besides, was lecturer on operative surgery at Saint George's Hospital. Mr. Herbert was the eldest son of Mr. William Allingham, whose treatise on diseases of the rectum has become a classic and which, in its later editions, bore the stamp of joint authorship by father and son.

Mr. Herbert Allingham at the time of his death and for some years previously bore the reputation, modestly, scientifically and justly acquired, of being one of England's most eminent surgeons. He was a teacher of great forcefulness and an author whose writings will stand in history.

